# Oxazolidinone resistance genes in florfenicol-resistant enterococci from beef cattle and veal calves at slaughter

**DOI:** 10.3389/fmicb.2023.1150070

**Published:** 2023-06-14

**Authors:** Magdalena Nüesch-Inderbinen, Michael Biggel, Adrian Haussmann, Andrea Treier, Lore Heyvaert, Nicole Cernela, Roger Stephan

**Affiliations:** ^1^Institute for Food Safety and Hygiene, Vetsuisse Faculty, University of Zurich, Zurich, Switzerland; ^2^Department of Veterinary and Biosciences, Faculty of Veterinary Medicine, University of Ghent, Ghent, Belgium

**Keywords:** florfenicol, linezolid, *optrA*, *poxtA*, beef cattle, veal calves, *E. faecalis*, *E. faecium* clade A1

## Abstract

**Background:**

Linezolid is a critically important oxazolidinone antibiotic used in human medicine. Although linezolid is not licensed for use in food-producing animals, the use of florfenicol in veterinary medicine co-selects for oxazolidinone resistance genes.

**Objective:**

This study aimed to assess the occurrence of *cfr, optrA*, and *poxtA* in florfenicol-resistant isolates from beef cattle and veal calves from different herds in Switzerland.

**Methods:**

A total of 618 cecal samples taken from beef cattle and veal calves at slaughter originating from 199 herds were cultured after an enrichment step on a selective medium containing 10 mg/L florfenicol. Isolates were screened by PCR for *cfr, optrA*, and *poxtA* which are genes known to confer resistance to oxazolidinones and phenicols. One isolate per PCR-positive species and herd was selected for antimicrobial susceptibility testing (AST) and whole-genome sequencing (WGS).

**Results:**

Overall, 105 florfenicol-resistant isolates were obtained from 99 (16%) of the samples, corresponding to 4% of the beef cattle herds and 24% of the veal calf herds. Screening by PCR revealed the presence of *optrA* in 95 (90%) and *poxtA* in 22 (21%) of the isolates. None of the isolates contained *cfr*. Isolates included for AST and WGS analysis were *Enterococcus* (*E*.) *faecalis* (*n* = 14), *E. faecium* (*n* = 12), *E. dispar* (*n* = 1), *E. durans* (*n* = 2), *E. gallinarum* (*n* = 1), *Vagococcus* (*V*.) *lutrae* (*n* = 2), *Aerococcus* (*A*.) *urinaeequi* (*n* = 1), and *Companilactobacillus* (*C*.) *farciminis* (*n* = 1). Thirteen isolates exhibited phenotypic linezolid resistance. Three novel OptrA variants were identified. Multilocus sequence typing identified four *E. faecium* ST18 belonging to hospital-associated clade A1. There was a difference in the replicon profile among *optrA-* and *poxtA*-harboring plasmids, with rep9 (RepA_*N*) plasmids dominating in *optrA*-harboring *E. faecalis* and rep2 (Inc18) and rep29 (Rep_3) plasmids in *poxtA*-carrying *E. faecium*.

**Conclusion:**

Beef cattle and veal calves are reservoirs for enterococci with acquired linezolid resistance genes *optrA* and *poxtA*. The presence of *E. faecium* ST18 highlights the zoonotic potential of some bovine isolates. The dispersal of clinically relevant oxazolidinone resistance genes throughout a wide variety of species including *Enterococcus* spp., *V. lutrae, A. urinaeequi*, and the probiotic *C. farciminis* in food-producing animals is a public health concern.

## 1. Introduction

The gastrointestinal tract of beef cattle and veal calves is reportedly a reservoir of antibiotic-resistant bacteria and clinically important antimicrobial resistance genes (Cameron and McAllister, 2016). Antimicrobial resistance (AMR) in food animals is thought to be promoted by antimicrobial use which is particularly high in the veal production sector (Rell et al., 2020; Becker et al., 2022). In beef cattle and veal calves, antimicrobial treatment consists of standard antimicrobial agents (aminoglycosides, β-lactams, diaminopyrimidines, phenicols, sulfonamides, and tetracyclines) depending on symptoms and body site of infection and restricted compounds which are classified as critically important for human medicine (fluoroquinolones, macrolides, and third- or fourth-generation cephalosporins) (European Union, 2019; World Health Organization, 2019; Swiss Veterinary Society, 2022). Florfenicol is a fluorinated derivative of chloramphenicol that is exclusively used in veterinary medicine and was licensed in Europe in 1995 for the treatment of bovine respiratory diseases (Schwarz et al., 2004). In Switzerland, florfenicol is recommended by the Swiss Veterinary Society (SVS) and the Federal Food Safety and Veterinary Office (FSVO) as a first-line antibiotic for the treatment of bovine respiratory disease in calves, calf meningitis, infectious bovine keratoconjunctivitis (IBK; also known as pinkeye) in cattle, and as a second-line antibiotic (after tetracycline) for metaphylaxis in calves and for the treatment of otitis in calves (Swiss Veterinary Society, 2022). The use of florfenicol in food-producing animals has selected for florfenicol-resistant bacteria, for example, enterococci harboring the phenicol exporter genes *fexA* or *fexB*, the 23S rRNA methyltransferase gene *cfr*, and genes encoding ribosomal protection proteins *optrA* and *poxtA* (Schwarz et al., 2004). Importantly, *cfr, optrA*, and *poxtA* may confer resistance not only to phenicols but also to oxazolidinones, potentially resulting in cross-resistance between florfenicol which is used exclusively in animals, and linezolid which is a critically important antibiotic to treat severe infections in humans (Long et al., 2006; Antonelli et al., 2018; Wang et al., 2018). Thus, due to the possibility of zoonotic transmission of AMR bacteria and horizontal transfer of resistance determinants to pathogenic strains within the human gut, the use of florfenicol in food-producing animals, including beef cattle and veal calves, may have serious consequences for public health. For beef, in particular, a possible transmission route may be through the consumption of undercooked or raw meat (rare steak or beef tartare). Linezolid-resistant enterococci from food-producing cattle have been described in Europe recently (de Jong et al., 2018; Timmermans et al., 2022), but data from beef cattle and veal calves in Switzerland are lacking. Therefore, this study was designed to (i) assess the occurrence of florfenicol resistance in presumptive enterococci from beef cattle and veal calves at slaughter in Switzerland using microbiological screening methodologies combined with matrix-assisted laser desorption/ionization time-of-flight mass spectrometry (MALDI–TOF MS) for species identification, (ii) identify any oxazolidinone resistance genes among the isolates using conventional polymerase chain reaction (PCR) protocols, and (iii) characterize a representative subset of the isolates by evaluating phenotypic AMR and applying whole-genome sequencing (WGS) analyses.

## 2. Materials and methods

### 2.1. Sampling and screening for florfenicol-resistant bacteria

A total of 618 cecal samples (360 from beef cattle and 258 from veal calves) from 199 herds (106 cattle beef herds and 93 calf veal herds) were collected between September and October 2021 at a major abattoir in Zürich, Switzerland. Given that herd size varied widely (1–19 animals per herd), samples were obtained from all animals from each herd processed during the sampling period, excluding any milk-producing cows or breeding bulls within a herd. Cecal contents were obtained from each animal immediately after slaughter by cutting through the cecal wall with sterile scissors. Each cecum was swabbed once to retrieve content, and each swab was placed in a sterile blender bag (Seward, Worthing, UK). Samples were transported to the laboratory in cooler boxes and processed on the same day. For each swab specimen, 50-ml brain heart infusion (BHI) broth with 6% NaCl (Oxoid, Pratteln, Switzerland) was added directly to each bag. The samples were homogenized using a Stomacher^®^ laboratory blender (Seward, Worthing, UK) and incubated for 18–24 h at 37°C. One loopful of the enriched culture was streaked on Bile Esculin Azide (BAA) agar (Merck, Darmstadt, Germany), supplemented with 10 mg/L florfenicol (Sigma-Aldrich, Buchs, Switzerland), and incubated under aerobic conditions for 48 h at 37°C. All bacterial colonies with morphological characteristics similar to *Enterococcus* spp. (small transparent colonies with brown-black halos) were subcultured on BAA agar with 10 mg/L florfenicol for 48 h at 37°C, and single colonies were subcultured on sheep blood agar (Difco, Becton Dickinson, Allschwil, Switzerland) for 24 h at 37°C. When morphologically different colonies were observed, both observed representative colonies were subcultured separately.

### 2.2. Species identification and screening for oxazolidinone resistance genes

The isolates were subjected to species identification using MALDI-TOF-MS (Bruker Daltonics, Billerica, MA, USA) and the Compass FlexControl version 3.4 software with the Compass database version 4.1.80. The presence of *optrA* and *poxtA* was established by singleplex PCR using custom synthesized primers (Microsynth, Balgach, Switzerland) and conditions described previously (Wang et al., 2015; Egan et al., 2020b). The *cfr* gene was searched using primer and conditions described earlier (Kehrenberg and Schwarz, 2006; Nüesch-Inderbinen et al., 2022a). All the isolates were stored in 20% glycerol at −20°C until further analysis.

One isolate per PCR-positive species and herd was selected for antimicrobial susceptibility testing (AST) and whole-genome sequencing (WGS). The isolates were selected based on the following criteria: (i) They represented various bacterial species in proportion to their occurrence [*Aerococcus urinaeequi, Companilactobacillus farciminis, Enterococcus (E.) dispar, E. faecalis, E. faecium, E. gallinarum*, and *Vagococcus lutrae*] and (ii) they were collected from animals originating from different herds.

### 2.3. Antimicrobial susceptibility testing

Antimicrobial susceptibility tests were performed using the agar disk diffusion method according to the guidelines of the Clinical and Laboratory Standards Institute (CLSI, 2023). Antimicrobial substances included chloramphenicol and linezolid (Becton, Dickinson, Heidelberg, Germany). *E. faecalis* ATCC 29212 was used as a quality control strain. Zone diameters were interpreted according to the CLSI (2023). Zone diameter breakpoints were ≤ 12 mm for chloramphenicol and ≤ 20 mm for linezolid, respectively. For bacterial genera where interpretive criteria for susceptibility testing are not available, the CLSI classification was not applied.

### 2.4. Whole-genome sequencing and genome analysis

Whole-genome sequences were determined using short-read sequencing (Illumina MiniSeq, Illumina, San Diego, CA, USA). Additionally, isolates containing plasmidal *optrA* or *poxtA* genes were long-read sequenced on a MinION Mk1B device (Oxford Nanopore Technologies, Oxford, UK). All isolates were first grown on sheep blood agar (Difco, Becton Dickinson, Allschwil, Switzerland), and genomic DNA was extracted using the DNeasy Blood and Tissue Kit (Qiagen, Hombrechtikon, Switzerland). Short-read sequencing libraries were prepared using the Nextera DNA Flex Library Preparation Kit (Illumina, San Diego, CA, USA) and sequenced on an Illumina MiniSeq instrument with 2 × 150 bp paired-end chemistries. For long-read sequencing, DNA was isolated using the MasterPure Complete DNA and RNA Purification Kit (Lucigen), and libraries were prepared using an SQK-LSK109 Ligation Sequencing Kit (Oxford Nanopore Technologies, Oxford, UK). The sequencing was carried out on a MinION Mk1B device using the FLO-MIN106 (R9) flow cell (Oxford Nanopore Technologies, Oxford, UK). Basecalling, demultiplexing, and barcode trimming were performed with guppy v4.2.2 (Oxford Nanopore Technologies, Oxford, UK) and quality-assessed with LongQC v1.2.0 (Fukasawa et al., 2020).

Short-reads from Illumina underwent assembly using SPAdes v.3.14.1 (Bankevich et al., 2012) implemented in Shovill v1.1.0 (Seemann, 2019). Hybrid assemblies were generated with Unicycler v.0.5.0 (Wick et al., 2017) using default settings. Genes were annotated using Bakta v1.6.1 (Schwengers et al., 2021), and isolates were typed *in silico* using mlst v.2.22.0 (Seemann, 2021b). For isolates that could not be definitively identified by MALDI-TOF MS, WGS was conducted to confirm the bacterial species. For species that could not be definitively identified by MALDI-TOF MS, genome assemblies were typed using the Ribosomal Multilocus Sequence Typing (rMLST) database (https://pubmlst.org/species-id) (Jolley et al., 2012).

Clade affiliation of *E. faecium* was determined using tools for the phylogenetic analysis described by Gouliouris et al. (2021) and phylogenetic clustering with 50 publicly available genomes for which clades were previously assigned (Lebreton et al., 2013). Following core genome alignment using Snippy v.4.6.0 (Seemann, 2015) with the chromosome of *E. faecium* SRR24 (GCF_009734005.1) as reference and masking of recombinant regions detected with Gubbins v.2.4.1 (Croucher et al., 2015), clusters were defined from the SNP alignment using fastbaps v.1.0.5 (Tonkin-Hill et al., 2019).

Antimicrobial resistance genes and plasmid replicons were identified using Abricate 1.0.1 (Seemann, 2021a) (70% coverage, 90% identity) in combination with the ResFinder 4.1 (Zankari et al., 2012) and PlasmidFinder databases (Carattoli et al., 2014), respectively. Plasmid replicon families were determined based on conserved domains identified in replicon sequences using BLAST (Madden, 2002). The *optrA* nucleotide sequences were compared with the wild-type (WT) *optrA*E349 (GenBank accession number KP399637) (Wang et al., 2015), and allele numbers of *optrA* were assigned according to the scheme from Freitas et al. (2020). OptrA variants were defined based on alterations in the deduced amino acid sequences. The nucleotide sequences of *poxtA* genes were compared to that described in *S. aureus* AOUC-0915 (GenBank accession no. MF095097) (Antonelli et al., 2018). Reads were also queried for mutations in the 23S rRNA genes associated with linezolid resistance (G2505A and G2576T) using LRE-finder 1.0 (Hasman et al., 2019).

### 2.5. Nucleotide accession numbers

Sequencing data and genome assemblies are available under BioProject no. PRJNA783265.

## 3. Results

### 3.1. Isolation of florfenicol-resistant bacteria and screening for oxazolidinone resistance determinants

Overall, 99 (16%) of the 618 cecal samples exhibited bacterial growth on BAA-florfenicol plates after 48 h, corresponding to 26 (13%) of the 199 herds. Among the 99 samples, six were from beef cattle (2% of the 360 beef cattle samples) and 93 from veal calves (36% of the 258 veal calf samples), corresponding to four (4%) of the 106 beef cattle herds and 22 (24%) of 93 the veal calf herds (Table 1). Six cecum samples yielded more than one distinct isolate, resulting in a total of 105 florfenicol-resistant strains (Table 1; Supplementary Table S1).

**Table 1:** Distribution of florfenicol-resistant isolates and oxazolidinone resistance genes among cecal samples from beef cattle and veal calves from 26 herds in Switzerland.

**Herd ID^a^ (size)**	**Positive samples (*n*)/isolates (*n*)**	**Bacterial species (*n*)**	***optrA/poxtA* gene profile (*n*)**
R39 (6)	2/2	*E. faecium (2)*	*optrA/poxtA (1), poxtA (1)*
R59 (1)	1/1	*E. durans (1)*	*poxtA (1)*
K68 (10)	1/1	*C. farciminis (1)*	*poxtA (1)*
K69 (2)	1/1	*E. faecium (1)*	*optrA/poxtA (1)*
K70 (13)	11/12	*E. faecalis (10)*	*optrA (10)*
		*E. faecium (2)*	*optrA/poxtA (2)*
K72 (5)	1/1	*E. faecalis (1)*	*optrA (1)*
K75 (1)	1/1	*E. faecium (1)*	*poxtA (1)*
K79 (1)	1/1	*A. urinaeequi (1)*	*optrA (1)*
K80 (17)	17/18	*E. faecalis (17)*	*optrA (17)*
		*E. faecium (1)*	*poxtA (1)*
K82 (18)	10/10	*E. faecalis (10)*	*optrA (10)*
K85 (3)	1/1	*E. faecalis (1)*	*optrA (1)*
R109 (2)	1/1	*E. durans (1)*	*poxtA (1)*
K136 (2)	1/1	*V. lutrae (1)*	*optrA (1)*
K137 (2)	2/2	*E. faecalis (2)*	*optrA (2)*
K162 (2)	2/2	*E. faecium (2)*	*optrA/poxtA (2)*
R186 (4)	2/2	*E. faecium (2)*	*poxtA (2)*
K188 (12)	10/10	*E. faecalis (9)*	*optrA (9)*
		*E. faecium (1)*	*optrA/poxtA (1)*
K189 (7)	4/4	*E. faecalis (3)*	*optrA (3)*
		*E. faecium (1)*	*poxtA (1)*
K190 (1)	1/1	*E. faecalis (1)*	*optrA (1)*
K191 (8)	8/9	*E. faecalis (6)*	*optrA (6)*
		*E. faecium (3)*	*optrA/poxtA (3)*
K192 (3)	1/1	*E. faecium (1)*	*poxtA (1)*
K194 (11)	7/7	*E. faecalis (7)*	*optrA (7)*
K195 (11)	9/11	*E. faecalis (9)*	*optrA (9)*
		*E. faecium (2)*	*optrA/poxtA (1), poxtA (1)*
K198 (2)	1/1	*E. faecalis (1)*	*optrA (1)*
K204 (1)	1/1	*V. lutrae*	*optrA (1)*
K205 (4)	2/3	*E. faecalis (1)*	*optrA (1)*
		*E. gallinarum (1)*	*optrA (1)*
		*E. dispar (1)*	*optrA (1)*

^a^*K* indicates veal calf herds and R signifies beef cattle herds.

Species identification revealed that *E. faecalis* (*n* = 78/105, 74%) was the predominant species, followed by *E. faecium* (*n* = 19/105, 18%), *E. durans* (*n* = 2/105, 2%), *E. dispar* (*n* = 1/105, 1%), and *E. gallinarum* (*n* = 1/105, 1%) (Table 1). Non-enterococcal species included *Vagococcus lutrae* (*n* = 2/105, 2%), *Aerococcus urinaeequi* (*n* = 1/105, 1%), and *Companilactobacillus farciminis* (*n* = 1/105, 1%) (Table 1).

PCR screening demonstrated the presence of oxazolidinone resistance genes in all 105 isolates (Table 1; Supplementary Table S1). The *optrA* gene was identified in 95 (90%) of the isolates, including *E. faecalis* (*n* = 78), *E. faecium* (*n* = 12), *E. dispar* (*n* = 1), *E. gallinarum* (*n* = 1), *V. lutrae* (*n* = 2), and *A. urinaeequi* (*n* = 1) (Table 1). The *poxtA* gene was present in a total of 22 (21%) of the isolates consisting of *E. faecium* (*n* = 19), *E. durans* (*n* = 2), and *C. farciminis* (*n* = 1). In 12 of the isolates, *poxtA* was co-harbored with *optrA* (Table 1). The *cfr* gene was not detected in any of the isolates (Supplementary Table S1).

For 19 of the 26 positive herds (herds R39, R59, K68, K69, K72, K75, K79, K82, K85, R109, K136, K137, K162, R186, K190, K192, K194, K198, and K204), all positive samples within each herd yielded the same bacterial species (Supplementary Table S1). Of these, one randomly selected isolate per herd was used for further analysis. For six herds (herds K70, K80, K188, K189, K191, and K195), two distinct bacterial species were isolated within each herd, and, in each case, one isolate of each species was included for subsequent analysis (Supplementary Table S1). Finally, one herd (K205) yielded samples containing three different species, all of which were included for further analysis (Supplementary Table S1). Thus, a total of 34 isolates from four cattle beef and from 30 veal calves were available for further characterization, including *E. faecalis* (*n* = 14), *E. faecium* (*n* = 12), *E. durans* (*n* = 2), *E. dispar* (*n* = 1), *E. gallinarum* (*n* = 1), *V. lutrae* (*n* = 2), *A. urinaeequi* (*n* = 1), and *C. farciminis* (*n* = 1). Thereof, *optrA* only was present in 19 isolates, *poxtA* only in 10, and *optrA*/*poxtA* in five isolates, respectively.

### 3.2. Antimicrobial susceptibility phenotypes

All 34 isolates were tested for their susceptibility to chloramphenicol and linezolid (Table 2). Resistance to chloramphenicol was noted for 23 (77%) of the enterococcal isolates, including *E. faecalis* (*n* = 12), *E. faecium* (*n* = 10), and *E. gallinarum* (*n* = 1). Resistance to linezolid was detected in 13 (43%) of the enterococcal isolates, including *E. faecalis* (*n* = 9), *E. faecium* (*n* = 3), and *E. gallinarum* (*n* = 1) (Table 2).

**Table 2:** WGS-based identification of resistance genes including linezolid-resistant genes in isolates from beef cattle and veal calves.

**Isolate ID**	**Species**	**MLST (clade)^a^**	**DD test (mm)^b^ C**	**DD test (mm)^b^ LDZ**	**Oxazolidinone resistance genes^c^**	**Other antimicrobial resistance genes**
K205-4b	*E. dispar*	–	14	22	*optrA13*	*erm*(B), *tet*(S)
R109-1	*E. durans*	–	13	27	*poxtA*	a*ac(6′)-Iih, fexB, tet*(L), *tet*(M)
R59-1	*E. durans*	–	13	22	*poxtA*	*aac(6′)-Iih, fexB*
K205-3	*E. faecalis*	19	14	**20**	*optrA1*	*ant(6)-Ia, aph(3′)-III, fexA, lsa*(A), *tet*(M)
K190-1	*E. faecalis*	25	14	23	*optrA8*	*aac(6′)-aph(2′'), erm*(B), *fexA, lsa*(A), *tet*(M)
K198-1	*E. faecalis*	234	**12**	**20**	*optrA2*	*erm*(B), *fexA, lsa*(A), *tet*(L), *tet*(M)
K188-1	*E. faecalis*	314	**11**	21	*optrA* ^*^	*aph(3′)-III, dfrG, erm*(A), *erm*(B), *fexA, lnu*(G), *lsa*(A), *tet*(L), *tet*(M)
K191-1	*E. faecalis*	314	**11**	**20**	*optrA* ^*^	*aph(3′)-III, dfrG, erm*(A), *erm*(B), *fexA, lnu*(G), *lsa*(A), *tet*(L), *tet*(M)
K72-1	*E. faecalis*	314	**8**	22	*optrA* ^*^	*aph(3′)-III, dfrG, erm*(A), *erm*(B), *fexA, lnu(*G), *lsa*(A), *tet*(L), *tet*(M)
K137-1a	*E. faecalis*	376	**8**	24	*optrA18*	*cat, erm*(B), *fexA*, lsa(A), *tet*(L), *tet*(M)
K194-1	*E. faecalis*	376	**8**	**20**	*optrA18*	*cat, erm*(B), *fexA*, lsa(A), *tet*(L), *tet*(M)
K70-12a	*E. faecalis*	376	**8**	**19**	*optrA18*	*cat, erm*(B), *fexA*, lsa(A), *tet*(L), *tet*(M)
K80-2b	*E. faecalis*	376	**8**	**20**	*optrA18*	*cat, erm*(B), *fexA*, lsa(A), *tet*(L), *tet*(M)
K162-1	*E. faecium*	38 (A2)	**8**	24	*optrA7, poxtA*	*aac(6′)-Ii, fexA, fexB, msr*(C), *tet*(L), *tet*(M)
K189-3	*E. faecium*	18 (A1)	**11**	25	*poxtA*	*aac(6′)-Ii, ant(6)-Ia, aph(3′)-III, dfrG, erm*(B), *fexB, msr*(C), *tet*(L), *tet*(M)
K70-1a	*E. faecium*	18 (A1)	**6**	21	*optrA11* (2x), *poxtA*	*aac(6′)-Ii, aac(6′)-aph(2′'), ant(6)-Ia, aph(3′)-III, dfrG, erm*(A), *erm*(B), *fexB, lnu*(B), *lsa*(E), *msr*(C), *tet*(L), *tet*(M)
K75-1	*E. faecium*	18 (A1)	14	26	*poxtA*	*aac(6′)-Ii, ant(6)-Ia, aph(3′)-III, dfrG, erm*(B)*, msr*(C), *tet*(L), *tet*(M)
K80-15b	*E. faecium*	18 (A1)	**12**	26	*poxtA*	*aac(6′)-Ii, ant(6)-Ia, aph(3′)-III, dfrG, erm*(B), *fexB, msr*(C), *tet*(L), *tet*(M)
K189-7	*E. faecalis*	593	**6**	26	*optrA19*	*ant(6)-Ia, aph(3′)-III, cat, dfrG, erm*(B), *fexA, lsa*(A), *str, tet*(L), *tet*(M)
K188-5	*E. faecium*	104 (A2)	**6**	**20**	*optrA1, poxtA*	*aac(6′)-Ii, dfrG, erm*(A), *erm*(B), *fexB, msr*(C), *tet*(L), *tet*(M)
K191-3	*E. faecium*	104 (A2)	**9**	**19**	*optrA1, poxtA*	*aac(6′)-Ii, dfrG, erm*(A), *erm*(B), *fexB, msr*(C), *tet*(L), *tet*(M)
K192-1	*E. faecium*	104 (A2)	**8**	25	*poxtA*	*aac(6′)-Ii, ant(6)-Ia, aph(3′)-III, erm*(B), *fexB, msr*(C), *tet*(L), *tet*(M)
K69-1a	*E. faecium*	104 (A2)	**8**	**19**	*optrA1, poxtA*	*aac(6′)-Ii, dfrG, erm*(A), *erm*(B), *fexB, msr*(C), *tet*(L), *tet*(M)
R186-2	*E. faecium*	324 (A2)	14	26	*poxtA*	*aac(6′)-Ii, erm*(B), *fexB, msr*(C), *tet*(L), *tet*(M)
R39-1	*E. faecium*	885 (A2)	**12**	25	*poxtA*	*aac(6′)-Ii, aac(6′)-aph(2′'), ant(6)-Ia, ant(9)-Ia, erm*(A), *erm*(B), *fexB, lnu*(B), *lsa*(E), *msr*(C), *tet*(L), *tet*(M)
K195-6b	*E. faecium*	2,103 (A2)	**9**	24	*poxtA*	*aac(6′)-Ii, aac(6′)-aph(2′'), dfrG, fexB, msr*(C)
K205-4a	*E. gallinarum*	–	**8**	**17**	*optrA* ^*^	*van*C1XY, *erm*(B), *fexA, tet*(M)
K195-1	*E. faecalis*	1,169	**6**	**19**	*optrA5*	*aac(6′)-aph(2′'), ant(9)-Ia, aph(3′)-III, cat, dfrG, erm*(A), *erm*(B), *fexA, lsa*(A), *tet*(L), *tet*(M)
K85-2	*E. faecalis*	1,169	**6**	**20**	*optrA5*	*aac(6′)-aph(2′'), ant(9)-Ia, aph(3′)-III, cat, dfrG, erm*(A), *erm*(B), *fexA, lsa*(A), *tet*(L), *tet*(M)
K82-1a	*E. faecalis*	1,169	**6**	**19**	*optrA5*	*aac(6′)-aph(2′'), ant(9)-Ia, aph(3′)-III, cat, dfrG, erm*(A), *erm*(B), *fexA, lsa*(A), *tet*(L), *tet*(M)
K79-1	*A. urinaeequi*	–	24	30	*optrA* ^*^	–
K68-8	*C. farciminis*	–	10	26	*poxtA*	*erm*(B), *fexB, tet*(M)
K136-2	*V. lutrae*	–	10	21	*optrA2*	*fexA, lnu(*G), *tet*(M)
K204-1	*V. lutrae*	–	11	30	*optrA2*	*ant(6)-Ia, aph(3′)-III, erm*(C), *erm*(Y), *fexA, lnu*(G), *tet*(M)

*A. urinaeequi, Aerococcus urinaeequi*; C, chloramphenicol; *C. farciminis, Companilactobacillus farciminis*; DD, disk diffusion test; LDZ, linezolid; MLST, multilocus sequence type; n.i., not identified; *V. lutrae, Vagococcus lutrae*; ^*^, novel *optrA* alleles; –, not applicable.

a Clades were determined for *E. faecium* (Lebreton et al., 2013).

b Zone diameter breakpoints for resistance were ≤ 12 mm for C and ≤ 20 for LDZ, according to the CLSI (2023). Zone diameter values interpreted as resistant are indicated in bold. *E. faecalis* ATCC 29212 was used as a quality control strain (zone diameter for C: 20 mm, zone diameter for LDZ: 24 mm). Breakpoints apply to *Enterococcus* spp. only.

c Allele numbering of *optrA* was carried out according to Freitas et al. (2020). The *optrA1* gene is identical to wild-type *optrA*_E349_ (GenBank accession number KP399637) (Wang et al., 2015).

Amino acid substitutions and their positions are shown in Table 3. Locations of the *optrA* and *poxtA* genes are listed in Table 4.

### 3.3. Genotyping of *E. faecalis* and *E. faecium*

Multilocus sequence typing analysis assigned the 14 *E. faecalis* to seven different sequence types (STs), namely, ST19 (*n* = 1), ST25 (*n* = 1), ST234 (*n* = 1), ST314 (*n* = 3), ST376 (*n* = 4), ST593 (*n* = 1), and ST1169 (*n* = 3) (Table 2). The 12 *E. faecium* belonged to six different STs, namely ST38 (*n* = 1), ST18 (*n* = 4), ST104 (*n* = 4), ST324 (*n* = 1), ST885 (*n* = 1), and ST2013 (*n* = 1). *E. faecium* ST18 was assigned to hospital-associated clade A1 and the remaining *E. faecium* STs to clade A2 (Table 2).

### 3.4. Identification of *optrA* and *poxtA* variants and other antimicrobial resistance genes

A WGS analysis identified 12 different *optrA* allelic variants (including the WT *optrA*_E349_, referred to in this study as *optrA1*) and three novel alleles in a total of 24 isolates. The corresponding amino acid sequences of the OptrA variants are summarized in Table 3. Among the isolates, *E. faecalis* ST19 (*n* = 1) and *E. faecium* ST104 (*n* = 3) harbored *optrA1* (GenBank acc. no. KP399637), *E. faecalis* ST234 (*n* = 1) and *V. lutrae* (*n* = 2) carried *optrA2* (GenBank acc. no. KT862781), *E. faecalis* ST1169 (*n* = 3) contained *optrA5* (GenBank acc. no. KT862783), *E. faecium* ST38 (*n* = 1) carried *optrA7* (GenBank acc. no. KT862784), *E. faecalis* ST25 (*n* = 1) contained *optrA8* (GenBank acc. no. KT862775), *E. faecium* ST18 (*n* = 1) harbored two copies of *optrA11* (GenBank acc. no. KT862782), *E. dispar* (*n* = 1) contained an *optrA13* (GenBank acc. no. KT892063), *E. faecalis* ST376 (*n* = 4) harbored *optrA18* (GenBank acc. no. KX620942), and *E. faecalis* ST593 (*n* = 1) contained *optrA19* (GenBank acc. no.KX620939). Three novel OptrA variants were found: *E. faecalis* ST314 (*n* = 3) harbored an *optrA* allelic variant encoding OptrA EK (Table 3). *E. gallinarum* (*n* = 1) and *A. urinaeequi* (*n* = 1) contained novel *optrA* alleles corresponding to variants OptrA EYDKWVDASKELYNKQLEIG, and OptrA EYKCLKVDASKELYNKQLEIG, respectively (Table 3).

**Table 3:** OptrA variants were detected among florfenicol-resistant isolates from beef cattle and veal calves.

***optrA* allele^a^**	**OptrA variant^b^**	**Amino acid substitution (s)^c^**	**Host species (*n*)**	**References**
*optrA1*	Wild-type OptrA_E349_	None	*E. faecalis* (1), *E. faecium* (3)	Wang et al., 2015
*optrA2*	EDM	K3**E**, Y176**D**, I622**M**	*E. faecalis* (1), *V. lutrae* (2)	He et al., 2016; Cai et al., 2019
*optrA5*	–	None	*E. faecalis* (3)	He et al., 2016; Freitas et al., 2020
*optrA7*	EDD	K3**E**, Y176**D**, G393**D**	*E. faecium (*1)	He et al., 2016; Cai et al., 2019
*optrA8*	DP	G40**D**, T481**P**	*E. faecalis* (1)	He et al., 2016; Freitas et al., 2020
*optrA11*	DD_3	Y176**D**, G393**D**	*E. faecium* (1)	He et al., 2016; Freitas et al., 2020
*optrA13*	EYKWDVDASKELYNKQLEIG	K3**E**, N12**Y**, N122**K**, Y135**W**, Y176**D**, A350**V**, G393**D**, V395**A**, A396**S**, Q509**K**, Q541**E**, M552**L**, N560**Y**, K562**N**, Q565**K**, E614**Q**, I627**L**, D633**E**, N640**I**, R650**G**	*E. dispar* (1)	Brenciani et al., 2019; Freitas et al., 2020
*optrA18*	RDK	I104**R**, Y176**D**, E256**K**	*E. faecalis* (4)	Cui et al., 2016; Freitas et al., 2020
*optrA19*	EYDNDM	K3**E**, N12**Y**, Y176**D**, D247**N**, G393**D**, I622**M**	*E. faecalis* (1)	Cui et al., 2016; Freitas et al., 2020
optrA^*^	EKD	K3**E**, T112**K**, Y176**D**	*E. faecalis* (3)	This study
optrA^*^	EYDKWDVDASKELYNKQLEIG	K3**E**, N12**Y**, G40**D**, N122**K**, Y135**W**, Y176**D**, A350**V**, G393**D**, V395**A**, A396**S**, Q509**K**, Q541**E**, M552**L**, N560**Y**, K562**N**, Q565**K**, E614**Q**, I627**L**, D633**E**, N640**I**, R650**G**	*E. gallinarum* (1)	This study
optrA^*^	EYKCLDKVDASKELYNKQLEIG	K3**E**, N12**Y**, N122**K**, Y135**C**, S147**L**, Y176**D**, L284**K**, A350**V**, G393**D**, V395**A**, A396**S**, Q509**K**, Q541**E**, M552**L**, N560**Y**, K562**N**, Q565**K**, E614**Q**, I627**L**, D633**E**, N640**I**, R650**G**	*A. urinaeequi (*1)	This study

* Novel *optrA* alleles and OptrA variants described in this study; –, not applicable.

a Allele numbering is according to Freitas et al. (2020). The *optrA1* gene is identical to wild-type *optrA*_E349_ (GenBank accession number KP399637) (Wang et al., 2015).

b Variant nomenclature is according to Schwarz et al. (2021).

c Amino acid sequences are compared with first-reported OptrA_E349_ from *E. faecalis* E349 (Wang et al., 2015). Substituted amino acids are shown in bold behind their positions within the sequence.

The WGS analysis identified the WT *poxtA* gene (GenBank accession no. MF095097) in *E. faecium* ST38 (*n* = 1), ST18 (*n* = 4), ST104 (*n* = 4), ST324 (*n* = 1), ST885 (*n* = 1), and ST2103 (*n* = 1), *E. durans* (*n* = 2), and *C. farciminis* (*n* = 1) (Table 2).

Almost all the isolates (*n* = 32, 94%) carried the phenicol resistance gene *fexA* (*n* = 17), *fexB* (*n* = 14), or *fexA* and *fexB* (*n* = 1) (Table 2). None of the isolates contained mutations in the 23S rRNA genes associated with linezolid resistance.

A number of additional resistance genes were identified, including genes conferring resistance to aminoglycosides [*aac(6*′*)-aph(2*′'*), aac(6*′*)-Ii, aac(6*′*)-Iih, ant(6)-Ia, ant(9)-Ia, ant(6)-Ia, aph(3*′*)-III*, and *str*], chloramphenicol (*cat*), lincosamides [*lnu*(G), *lsa*(A), *and lsa*(E)], macrolides [*erm*(A), *erm*(B), and *msr*(C)], tetracycline [*tet*(L) and *tet*(M)], trimethoprim (*dfrG*), and vancomycin (*van*C1XY) (Table 2).

Among the AMR genes, *lsa*(A) was found in every *E. faecalis* and *msr*(C) in every *E. faecium* isolate, respectively. Furthermore, *tet*(L) and/or *tet*(M) was identified in 31 (91%) of the isolates, and *E. gallinarum* (*n* = 1) harbored the vancomycin resistance gene *van*C1XY (Table 2). *A. urinaeequi* (isolate ID K79-1) did not carry any AMR genes apart from *optrA* (Table 2).

### 3.5. Location of *optrA* and *poxtA* genes

Overall, *optrA* was located within the chromosome (*n* = 5), on plasmids (*n* = 18), or both (*n* = 1) (Table 4). In *E. faecalis*, nine of the 10 identified *optrA*-encoding plasmids were categorized within the replicon type rep9a and rep9b belonging to the RepA_*N* family, with plasmid sizes ranging between 51.4 and 78.7 kb (Table 4). In *E. faecalis* ST376, *optrA* was associated with rep9a plasmids (*n* = 4), and in *E. faecalis*, ST25, ST234, and ST314, *optrA* were found on rep9b plasmids (*n* = 5). The remaining *E. faecalis* (ST19) harbored *optrA* on a 36.3 kb repUS40_1 Rep_3 family plasmid (Table 4).

**Table 4:** Location of *optrA* and *poxtA* genes among florfenicol-resistant isolates from beef cattle and veal calves.

				***optrA***			***poxtA***		
**Isolate ID**	**Species**	**MLST (clade)**	**Oxazolidinone resistance gene(s)**	**Location**	**Plasmid size (bp)**	**Replicon type [family]**	**Location**	**Plasmid size (bp)**	**Replicon type [family]**
K205-4b	*E. dispar*	–	*optrA13*	P	8,638	n.i.	–	–	–
R109-1	*E. durans*	–	*poxtA*	–	–	–	P	27,897	rep2_1 [Inc18]
R59-1	*E. durans*	–	*poxtA*	–	–	–	P	23,710	rep29_2 [Rep_3]
K205-3	*E. faecalis*	19	*optrA1*	P	36,331	repUS40_1 [Rep_3]	–	–	–
K190-1	*E. faecalis*	25	*optrA8*	P	63,994	rep9b_2 [RepA_N]	–	–	–
K198-1	*E. faecalis*	234	*optrA2*	P	78,685	rep9b_2 [RepA_N], repUS43_1 [Rep_trans]	–	–	–
K188-1	*E. faecalis*	314	optrA^*^	P	74,469	rep9b_2 [RepA_N]	–	–	–
K191-1	*E. faecalis*	314	optrA^*^	P	74,469	rep9b_2 [RepA_N]	–	–	–
K72-1	*E. faecalis*	314	optrA^*^	P	74,469	rep9b_2 [RepA_N]	–	–	–
K137-1a	*E. faecalis*	376	*optrA18*	P	51,389	rep9a_1 [RepA_N], repUS43_1 [Rep_trans]	–	–	–
K194-1	*E. faecalis*	376	*optrA18*	P	59,610	rep9a_1 [RepA_N], repUS43_1 [Rep_trans]	–	–	–
K70-12a	*E. faecalis*	376	*optrA18*	P	59,165	rep9a_1 [RepA_N], repUS43_1 [Rep_trans]	–	–	–
K80-2b	*E. faecalis*	376	*optrA18*	P	59,599	rep9a_1 [RepA_N], repUS43_1 [Rep_trans]	–	–	–
K162-1	*E. faecium*	38 (A2)	*optrA7, poxtA*	C	–	–	P	27,897	rep2_1 [Inc18]
K189-3	*E. faecium*	18 (A1)	*poxtA*	–	–	–	P	38,387	rep2_1 [Inc18]
K70-1a	*E. faecium*	18 (A1)	*optrA11, poxtA*	C + P	242,833	repUS15_2 [RepA_N]	P	39,454	rep2_1 [Inc18]
K75-1	*E. faecium*	18 (A1)	*poxtA*	–			P	38,387	rep2_1 [Inc18]
K80-15b	*E. faecium*	18 (A1)	*poxtA*	–	–	–	P	38,387	rep2_1 [Inc18]
K189-7	*E. faecalis*	593	*optrA19*	C					
K188-5	*E. faecium*	104 (A2)	*optrA1, poxtA*	P	184,843	repUS15_2 [RepA_N]	P	24,566	rep29_2 [Rep_3]
K191-3	*E. faecium*	104 (A2)	*optrA1, poxtA*	P	186,836	repUS15_2 [RepA_N]	P	24,768	rep29_2 [Rep_3]
K192-1	*E. faecium*	104 (A2)	*poxtA*	–			P	17,306^a^	rep29_2 [Rep_3]
K69-1a	*E. faecium*	104 (A2)	*optrA1, poxtA*	P	184,656	repUS15_2 [RepA_N]	P	28,578	rep29_2 [Rep_3]
R186-2	*E. faecium*	324 (A2)	*poxtA*	–	–	–	P	46,474	rep2_1 [Inc18]
R39-1	*E. faecium*	885 (A2)	*poxtA*	–	–	–	P	77,238	rep2_1 [Inc18], repUS43_1 [Rep_trans]
K195-6b	*E. faecium*	2103 (A2)	*poxtA*	–	–	–	P	44,010	rep14a_1 [Rep_trans]
K205-4a	*E. gallinarum*	–	optrA^*^	P	18,271	rep1_1 [Inc18]	–	–	–
K195-1	*E. faecalis*	1169	*optrA5*	C	–	–	–	–	–
K85-2	*E. faecalis*	1169	*optrA5*	C	–	–	–	–	–
K82-1a	*E. faecalis*	1169	*optrA5*	C	–	–	–	–	–
K79-1	*A. urinaeequi*	–	optrA^*^	P	29,310	n.i.	–	–	–
K68-8	*C. farciminis*	–	*poxtA*	–	–	–	C	–	–
K136-2	*V. lutrae*	–	*optrA2*	P	19,187	n.i.	–	–	–
K204-1	*V. lutrae*	–	*optrA2*	P	27,307	repUS12_3 [Rep_1]	–	–	–

C, chromosomal DNA; P, plasmid; –, gene not present;

* , novel *optrA* allele; n.i., not identifiable.

a Incompletely assembled; size based on assembly graph.

Among the *E. faecium* isolates, all *optrA*-carrying plasmids (*n* = 4) belonged to repUS15_2 (RepA_N) (Table 4). In *E. faecium* ST104, plasmid sizes (18.5 kb) were identical (*n* = 3). In *E. faecium* ST18, *optrA* was carried on a 24.3 kb repUS15_2 (RepA_N) plasmid (Table 4). In *E. gallinarum, optrA* was identified on an 18.3 kb rep1_1 (Inc18) plasmid, and *V. lutrae* carried *optrA* on a 27.3 kb repUS12_3 (Rep_1) plasmid (Table 4).

PlasmidFinder (Carattoli et al., 2014) did not identify *rep* genes in a total of three *optrA*-encoding plasmids from *A. urinaeequi, E. dispar*, and *V. lutrae* (Table 4).

The *poxtA* genes were located within the chromosome (*n* = 1) and on plasmids (*n* = 14).

Among *E. faecium* ST18, ST38, ST324, and ST885, *poxtA* was identified on rep2_1 plasmids (*n* = 7) belonging to the Inc18 family, ranging between 27.9 and 77.2 kb in size (Table 4). In *E. faecium* ST104, *poxtA* was found on 17.3–12.5 kb rep29_2 (Rep_3) plasmids (*n* = 4), and in *E. faecium* ST 2103, *poxtA* was located on a 44 kb rep14a_1 (Rep_trans) plasmid (*n* = 1) (Table 4). In *E. durans, poxtA* was identified on a 27.9 kb rep2_1 (Inc18) (*n* = 1) and a 23.7kb rep29_2 (Rep_3) plasmid (*n* = 1), respectively (Table 4).

## 4. Discussion

In this study, we found the presence of florfenicol-resistant *Enterococcus*-like bacteria in 16% of cecal samples of healthy beef cattle and veal calves in Switzerland. Our data show that the occurrence of florfenicol-resistant isolates differed widely between beef cattle (2%) and veal calves (36%), corresponding to a herd prevalence of 4% for beef cattle and 24% for veal calves, respectively. These results may reflect the high usage of antimicrobials in Swiss calf fattening operations (Becker et al., 2022), which, for its part, is mainly due to the predisposition to infectious diseases of young calves challenged by transportation, co-mingling, and inadequate housing (Becker et al., 2020). In addition, most isolates analyzed in this study were associated with the presence of tetracycline resistance genes. Tetracyclines are widely used for the treatment of bovine infectious diseases and metaphylaxis (Swiss Veterinary Society, 2022) and are, therefore, similar to florfenicol, likely to further promote the spread and persistence of bacteria that harbor oxazolidinone resistance genes. In this study, all 105 florfenicol-resistant isolates carried oxazolidinone resistance genes. Notably, for many of the isolates, the presence of these genes alone did not result in phenotypic resistance. This observation corroborates previous studies that describe varying linezolid MICs among *Enterococcus* spp., their STs, and their *optrA* or *poxtA* genes (Wang et al., 2015; Cai et al., 2019; Nüesch-Inderbinen et al., 2022a). While bacterial host factors are suggested to influence the expression of linezolid resistance genes (Cai et al., 2019), the mechanisms that determine the resistance phenotype of Gram-positive bacteria harboring linezolid resistance determinants remain unclear. The lack of a linezolid resistance phenotype among many isolates in this study indicates that our selective approach using florfenicol to detect linezolid resistance mechanisms in enterococci and other bacterial genera may be favorable for screening samples from food-producing animals.

The majority (76%) of the florfenicol-resistant isolates were represented by *E. faecalis* or *E. faecium*. These two enterococcal species are part of the normal gastrointestinal flora in animals and humans, but they also belong to the most important nosocomial pathogens worldwide (Aarestrup, 2015; Zaheer et al., 2020). Among the isolates collected during this study, WGS analysis identified *E. faecalis* ST19 (isolate ID K205-3)-harboring plasmid-borne *optrA1*. Isolate K205-3 represented one of the 13 phenotypically linezolid-resistant isolates identified in this study according to CLSI definitions. The *optrA1* gene belongs to one of the most widespread variants identified among *optrA*-positive enterococci from food-producing animals and humans worldwide (Wang et al., 2015). *E. faecalis* ST19-harboring *optrA1* has also been isolated from a hospitalized patient in Ireland (Egan et al., 2020b). Similarly, *E. faecalis* ST593 carrying *optrA19* (Optra EYDNDM) identified in one veal calf in this study has been associated with urinary tract infection in humans (Cai et al., 2019), highlighting the potential of *optrA*-harboring *E. faecalis* occurring in food-producing animals to cause disease in humans.

WGS analysis further identified four *E. faecalis* ST376 (isolates K137-1a, K194-1, K70-12a, and K80-2b, recovered from calves across different herds) harboring plasmid-encoded *optrA18* (OptrA RDK), a globally widespread *optrA* variant originally described in human clinical *E. faecalis* (Cui et al., 2016). *E. faecalis* ST376 carrying *optrA18* has been found in fecal samples of healthy adults and children in China, in fresh commercial flowers in China, and in a cecal isolate from a fattening pig in Switzerland (Cai et al., 2019; Yu et al., 2021; Nüesch-Inderbinen et al., 2022a). The detection of *optrA18*-positive *E. faecalis* ST376 among bovine enterococci across different herds in the present study provides further evidence for its worldwide dissemination throughout multiple ecological niches.

WGS showed that the bovine *E. faecium* from this study included four *E. faecium* ST18 belonging to the epidemic hospital-associated lineage referred to as clade A1 (isolates K189-3, K70-1a, K75-1, and K80-15b, respectively) (Lebreton et al., 2013). Three of these isolates carried plasmid-mediated *poxtA* with the fourth additionally harboring a chromosomal *optrA*. Similar linezolid-resistant *E. faecium* ST18 strains carrying plasmid-mediated *poxtA* with or without chromosomal *optrA* have been recovered from hospitalized patients in Ireland between 2016 and 2019 (Egan et al., 2020a). In addition, *optrA*-harboring *E. faecalis* ST18 has been found in fecal samples from veal calves in Belgium in 2019 (Timmermans et al., 2022), emphasizing the dispersal of *poxtA* and/or *optrA*-positive *E. faecium* ST18 across different hosts and regions. The actual risk of zoonotic transmission of *Enterococcus* spp. from livestock to humans is probably quite low (Gouliouris et al., 2018; Cattoir, 2022). Furthermore, our data are not discriminative enough to infer a strong relatedness between clinical and livestock isolates; nevertheless, it cannot be excluded that the occurrence of *E. faecium* ST18 in veal calves may represent a potential zoonotic risk. Additional screening of the employees in the slaughterhouse may have provided indications of possible transmission events; however, this was not performed in this study.

The remaining *E. faecium* analyzed in this study were part of clade A2, which is mostly represented by strains from animal commensals (Lebreton et al., 2013). Isolates from clade A2 included *optrA1*/*poxtA-*carrying *E. faecium* ST104 collected across four veal calf herds, which has previously been found in fecal samples from veal calves in Belgium (Timmermans et al., 2022).

Apart from *E. faecalis* and *E. faecium*, we detected plasmid-encoded *optrA* in *E. dispar, E. durans*, and *E. gallinarum*, demonstrating a high diversity among *optrA* and/or *poxtA*-carrying enterococci circulating among beef cattle and veal calf herds in Switzerland. *E. gallinarum* harboring *van*C1XY and *optrA2* (OptrA EDM) was previously recovered from a porcine fecal swab in China in 2018 (Yao et al., 2020), and *E. gallinarum* co-harboring *van*C1XY and OptrA (KLDP) was found recently in healthy humans in Switzerland in 2021 (Nüesch-Inderbinen et al., 2022b), but this is to our knowledge the first description of a bovine *E. gallinarum* carrying *van*C1XY and a novel *optrA* (OptrA EYDKWDVDASKELYNKQLEIG) variant. Although *E. gallinarum* harboring *van*C1XY has been isolated from a patient with sepsis in Malaysia in 2019 (Mastor et al., 2020), this species is generally not considered pathogenic to humans, and its zoonotic potential is probably low (Cattoir, 2022).

In this study, *optrA* and *poxtA* were distributed unevenly among the enterococcal species and plasmid families, with most *optrA* found in *E. faecalis* and identified on rep9 variants of RepA_N plasmids which are narrow host range plasmids considered specific for *E. faecalis* (Mikalsen et al., 2015). By contrast, in *optrA*-harboring *E. faecium*, the repUS15_2 was the predominant replicon type but was absent in *E. faecalis*. Furthermore, plasmid-encoded *poxtA* was detected predominantly among *E. faecium* and found on broad host range rep2 (Inc18) and on narrow host range rep29 (Rep_3) plasmids which were lacking in *E. faecalis*, comparable to previous observations (Mikalsen et al., 2015). Taken together, our findings indicate that the dissemination of oxazolidinone resistance genes among bovine isolates is driven by different plasmid families in *E. faecalis* and *E. faecium*, similar to the development observed in clinically relevant enterococcal lineages (Mikalsen et al., 2015).

Finally, this study revealed the presence of *optrA* and *poxtA* in several non-enterococcal isolates, including *V. lutrae, A. urinaeequi*, and *C. farciminis*. While *optrA-*positive *V. lutrae* and *optrA*-harboring *A. urinaeequi* have previously been isolated from porcine samples in China (Yang et al., 2021; Zhu et al., 2022a), this is only the second report of *optrA* in *A. urinaeequ*i and the first description of a novel, plasmid-encoded *optrA* variant encoding OptrA EYKCLKVDASKELYNKQLEIG.

Furthermore, while *poxtA* has been identified in Lactobacillaceae, e.g., in *L. salivarius* recovered from human and porcine samples in China (Shen et al., 2022; Zhu et al., 2022b), to the best of our knowledge, this is the first report of *poxtA* in *C. farciminis* [formerly *L. farciminis* (Zheng et al., 2020)]. *C. farciminis* is authorized and presumed safe as a probiotic feed additive for animals in the EU (Directive 70/524/EEC, JLO 297:15/11/2001) (Coeuret et al., 2004; EFSA, 2021). The identification in this study of *poxtA*-carrying *C. farciminis* notably co-harboring *erm*(B), *fexB*, and *tet*(M) points to the possibility of transmission of AMR genes within the bovine intestine and raises questions regarding the role of probiotics as reservoirs of transferable AMR genes.

## 5. Conclusion

Our study shows that oxazolidinone resistance determinants occur in a variety of florfenicol-resistant *Enterococcus* spp., in *V. lutrae, A. urinaeequi*, and the probiotic *C. farciminis*, within the intestinal tract of beef cattle and veal calves. A potential health hazard is highlighted by the presence of *poxtA* and *poxtA*/*optrA*-harboring *E. faecium* ST18, which is associated with nosocomial infections. Co-selection of oxazolidinone resistance genes through the use of florfenicol represents a serious threat to the efficacy of linezolid and represents an issue that should be considered using a one-health approach.

## Data availability statement

The data presented in this study are deposited in the NCBI repository, accession number PRJNA783265: https://www.ncbi.nlm.nih.gov/bioproject/PRJNA783265.

## Ethics statement

No animal was killed for the purpose of providing samples. Ethics approval was not required for this study. All information was treated anonymously.

## Author contributions

The study was designed by RS. Sampling was accomplished by AH. Microbiological analyses were performed by AH, AT, and LH. WGS was done by MB and NC. Bioinformatics analyses were conducted by MB. Data analyses were conducted by AH, MB, MN-I, and RS. The manuscript was written by MN-I. RS and MB contributed to writing and revising the manuscript. All authors agreed on the final version. All authors contributed to the article and approved the submitted version.
