# Supplementary material for: Oxazolidinone resistance genes in florfenicol-resistant enterococci from beef cattle and veal calves at slaughter
Source: Front Microbiol. 2023 Jun 14;14:1150070. doi: 10.3389/fmicb.2023.1150070 (PMC10301837; doi:10.3389/fmicb.2023.1150070)
Supplement: Supplementary file 1 [file Table_1.DOCX]

Supplementary Material

Oxazolidinone resistance genes in florfenicol resistant enterococci from beef cattle and veal calves at slaughter

**Magdalena Nüesch-Inderbinen*, Michael Biggel, Adrian Haussmann, Andrea Treier, Lore Heyvaert, Nicole Cernela, Roger Stephan**

*** Correspondence:** Magdalena Nüesch-Inderbinen: magdalena.nueesch-inderbinen@uzh.ch

# Supplementary Table

Table S1. Distribution of florfenicol-resistant isolates and oxazolidinone resistance genes among cecal samples from beef cattle and veal calves from 26 herds in Switzerland.

|  |  |  |  |  | **Oxazolidinone resistance gene^b^** | | |
| --- | --- | --- | --- | --- | --- | --- | --- |
| **Herd ID^a^** | **Sample size (n)** | **Positive samples (n)** | **Isolate ID** | **Species** | ***cfr*** | ***optrA*** | ***poxtA*** |
| R39 | 6 | 2 | R39-1 | *E. faecium* | - | - | + |
| R39 | 6 |  | R39-3 | *E. faecium* | - | + | + |
| R59 | 1 | 1 | R59-1 | *E. durans* | - | - | + |
| K68 | 10 | 1 | K68-8 | *C. farciminis* | - |  | + |
| K69 | 2 | 1 | K69-1a | *E. faecium* | - | + | + |
| K70 | 13 | 11 | K70-1 | *E. faecium* | - | + | + |
| K70 | 13 |  | K70-2 | *E. faecalis* | - | + | - |
| K70 | 13 |  | K70-3 | *E. faecalis* | - | + | - |
| K70 | 13 |  | K70-4 | *E. faecalis* | - | + | - |
| K70 | 13 |  | K70-5 | *E. faecalis* | - | + | - |
| K70 | 13 |  | K70-6 | *E. faecalis* | - | + | - |
| K70 | 13 |  | K70-7a | *E. faecium* | - | + | + |
| K70 | 13 |  | K70-7b | *E. faecalis* | - | + | - |
| K70 | 13 |  | K70-9 | *E. faecalis* | - | + | - |
| K70 | 13 |  | K70-11 | *E. faecalis* | - | + | - |
| K70 | 13 |  | K70-12 | *E. faecalis* | - | + | - |
| K70 | 13 |  | K70-13 | *E. faecalis* | - | + | - |
| K72 | 5 | 1 | K72-1 | *E. faecalis* | - | + | - |
| K75 | 1 | 1 | K75-1 | *E. faecium* | - | - | + |
| K79 | 1 | 1 | K79-1 | *A. urinaeequi* | - | + |  |
| K80 | 17 | 18 | K80-1 | *E. faecalis* | - | + | - |
| K80 | 17 |  | K80-2 | *E. faecalis* | - | + | - |
| K80 | 17 |  | K80-3 | *E. faecalis* | - | + | - |
| K80 | 17 |  | K80-4 | *E. faecalis* | - | + | - |
| K80 | 17 |  | K80-5 | *E. faecalis* | - | + | - |
| K80 | 17 |  | K80-6 | *E. faecalis* | - | + | - |
| K80 | 17 |  | K80-7 | *E. faecalis* | - | + | - |
| K80 | 17 |  | K80-8 | *E. faecalis* | - | + | - |
| K80 | 17 |  | K80-9 | *E. faecalis* | - | + | - |
| K80 | 17 |  | K80-10 | *E. faecalis* | - | + | - |
| K80 | 17 |  | K80-11 | *E. faecalis* | - | + | - |
| K80 | 17 |  | K80-12 | *E. faecalis* | - | + | - |
| K80 | 17 |  | K80-13 | *E. faecalis* | - | + | - |
| K80 | 17 |  | K80-14 | *E. faecalis* | - | + | - |
| K80 | 17 |  | K80-15a | *E. faecalis* | - | + | - |
| K80 | 17 |  | K80-15b | *E. faecium* | - | + | + |
| K80 | 17 |  | K80-16 | *E. faecalis* | - | - | - |
| K80 | 17 |  | K80-17 | *E. faecalis* | - | + | - |
| K82 | 18 | 10 | K82-1 | *E. faecalis* | - | + | - |
| K82 | 18 |  | K82-3 | *E. faecalis* | - | + | - |
| K82 | 18 |  | K82-5 | *E. faecalis* | - | + | - |
| K82 | 18 |  | K82-6 | *E. faecalis* | - | + | - |
| K82 | 18 |  | K82-7 | *E. faecalis* | - | + | - |
| K82 | 18 |  | K82-8 | *E. faecalis* | - | + | - |
| K82 | 18 |  | K82-11 | *E. faecalis* | - | + | - |
| K82 | 18 |  | K82-12 | *E. faecalis* | - | + | - |
| K82 | 18 |  | K82-15 | *E. faecalis* | - | + | - |
| K82 | 18 |  | K82-16 | *E. faecalis* | - | + | - |
| K85 | 3 | 1 | K85-2 | *E. faecalis* | - | + | - |
| R109 | 2 | 1 | R109-1 | *E. durans* | - | - | + |
| K136 | 2 | 1 | K136-2 | *V. lutrae* | - | + |  |
| K137 | 2 | 2 | K137-1 | *E. faecalis* | - | + | - |
| K137 | 2 |  | K137-2 | *E. faecalis* | - | + | - |
| K162 | 2 | 2 | K162-1 | *E. faecium* | - | + | + |
| K162 | 2 |  | K162-2 | *E. faecium* | - | + | + |
| R186 | 4 | 2 | R186-2 | *E. faecium* | - | - | + |
| R186 | 4 |  | R186-4 | *E. faecium* | - | - | + |
| K188 | 12 | 10 | K188-1 | *E. faecalis* | - | + | - |
| K188 | 12 |  | K188-2 | *E. faecalis* | - | + | - |
| K188 | 12 |  | K188-3 | *E. faecalis* | - | + | - |
| K188 | 12 |  | K188-5 | *E. faecium* | - | + | + |
| K188 | 12 |  | K188-6 | *E. faecalis* | - | + | - |
| K188 | 12 |  | K188-7 | *E. faecalis* | - | + | - |
| K188 | 12 |  | K188-8 | *E. faecalis* | - | + | - |
| K188 | 12 |  | K188-10 | *E. faecalis* | - | + | - |
| K188 | 12 |  | K188-11 | *E. faecalis* | - | + | - |
| K188 | 12 |  | K188-12 | *E. faecalis* | - | + | - |
| K189 | 7 | 4 | K189-3 | *E. faecium* | - | - | + |
| K189 | 7 |  | K189-4 | *E. faecalis* | - | - | - |
| K189 | 7 |  | K189-6 | *E. faecalis* | - | + | - |
| K189 | 7 |  | K189-7 | *E. faecalis* | - | + | - |
| K190 | 1 | 1 | K190-1 | *E. faecalis* | - | + | - |
| K191 | 8 | 8 | K191-1 | *E. faecalis* | - | + | - |
| K191 | 8 |  | K191-2 | *E. faecium* | - | + | + |
| K191 | 8 |  | K191-3 | *E. faecium* | - | + | + |
| K191 | 8 |  | K191-4a | *E. faecium* | - | + | + |
| K191 | 8 |  | K191-4b | *E. faecalis* | - | + | - |
| K191 | 8 |  | K191-5 | *E. faecalis* | - | + | - |
| K191 | 8 |  | K191-6 | *E. faecalis* | - | + | - |
| K191 | 8 |  | K191-7 | *E. faecalis* | - | + | - |
| K191 | 8 |  | K191-8 | *E. faecalis* | - | + | - |
| K192 | 3 | 1 | K192-1 | *E. faecium* | - | - | + |
| K194 | 11 | 7 | K194-1 | *E. faecalis* | - | + | - |
| K194 | 11 |  | K194-4 | *E. faecalis* | - | + | - |
| K194 | 11 |  | K194-5 | *E. faecalis* | - | + | - |
| K194 | 11 |  | K194-7 | *E. faecalis* | - | + | - |
| K194 | 11 |  | K194-8 | *E. faecalis* | - | + | - |
| K194 | 11 |  | K194-10 | *E. faecalis* | - | + | - |
| K194 | 11 |  | K194-11 | *E. faecalis* | - | + | - |
| K195 | 11 | 9 | K195-1 | *E. faecalis* | - | + | - |
| K195 | 11 |  | K195-2 | *E. faecalis* | - | + | - |
| K195 | 11 |  | K195-3 | *E. faecalis* | - | + | - |
| K195 | 11 |  | K195-6a | *E. faecalis* | - | + | - |
| K195 | 11 |  | K195-6b | *E. faecium* | - | - | + |
| K195 | 11 |  | K195-7a | *E. faecalis* | - | + | - |
| K195 | 11 |  | K195-7b | *E. faecium* | - | + | + |
| K195 | 11 |  | K195-8 | *E. faecalis* | - | + | - |
| K195 | 11 |  | K195-9 | *E. faecalis* | - | + | - |
| K195 | 11 |  | K195-10 | *E. faecalis* | - | + | - |
| K195 | 11 |  | K195-11 | *E. faecalis* | - | + | - |
| K198 | 2 | 1 | K198-1 | *E. faecalis* | - | + | - |
| K204 | 1 | 1 | K204-1 | *V. lutrae* | - | + |  |
| K205 | 4 | 2 | K205-3 | *E. faecalis* | - | + | - |
| K205 | 4 |  | K205-4a | *E. gallinarum* | - | + | - |
| K205 | 4 |  | K205-4b | *E. dispar* | - | + | - |

^a^ K indicates veal calf herds, R signifies beef cattle herds.

^b^ +, presence of the gene; -, absence of the gene, determined by PCR.
